# WASN-Based Day–Night Characterization of Urban Anomalous Noise Events in Narrow and Wide Streets

**DOI:** 10.3390/s20174760

**Published:** 2020-08-23

**Authors:** Francesc Alías, Joan Claudi Socoró, Rosa Ma Alsina-Pagès

**Affiliations:** GTM – Grup de recerca en Tecnologies Mèdia, La Salle – Universitat Ramon Llull. c/Quatre Camins, 30, 08022 Barcelona, Spain; joanclaudi.socoro@salle.url.edu (J.C.S.); rosamaria.alsina@salle.url.edu (R.M.A.-P.)

**Keywords:** noise events, database, acoustic analysis, wireless acoustic sensor networks, dynamic noise mapping, low-cost sensors, urban environment, day–night periods, narrow–wide streets

## Abstract

In addition to air pollution, environmental noise has become one of the major hazards for citizens, being Road Traffic Noise (RTN) as its main source in urban areas. Recently, low-cost Wireless Acoustic Sensor Networks (WASNs) have become an alternative to traditional strategic noise mapping in cities. In order to monitor RTN solely, WASN-based approaches should automatize the off-line removal of those events unrelated to regular road traffic (e.g., sirens, airplanes, trams, etc.). Within the LIFE DYNAMAP project, 15 urban Anomalous Noise Events (ANEs) were described through an expert-based recording campaign. However, that work only focused on the overall analysis of the events gathered during non-sequential diurnal periods. As a step forward to characterize the temporal and local particularities of urban ANEs in real acoustic environments, this work analyses their distribution between day (06:00–22:00) and night (22:00–06:00) in narrow (1 lane) and wide (more than 1 lane) streets. The study is developed on a manually-labelled 151-h acoustic database obtained from the 24-nodes WASN deployed across DYNAMAP’s Milan pilot area during a weekday and a weekend day. Results confirm the unbalanced nature of the problem (RTN represents 83.5% of the data), while identifying 26 ANE subcategories mainly derived from pedestrians, animals, transports and industry. Their presence depends more significantly on the time period than on the street type, as most events have been observed in the day-time during the weekday, despite being especially present in narrow streets. Moreover, although ANEs show quite similar median durations regardless of time and location in general terms, they usually present higher median signal-to-noise ratios at night, mainly on the weekend, which becomes especially relevant for the WASN-based computation of equivalent RTN levels.

## 1. Introduction

Nowadays, 55% of people is living in urban areas, a percentage that is expected to grow to around 70% by 2050 according to the United Nations [1]. As a consequence, urban areas should develop sustainably so as to guarantee the quality of life of their inhabitants by considering economic, social and environmental dimensions in an integrated manner [1]. Together with air pollution, environmental noise is increasing year after year, becoming one of the major hazards in populated areas [2,3]. Among the different noise sources, several studies have demonstrated the negative effects on people of Road Traffic Noise (RTN), as the main noise source in cities (e.g., see [4,5]). RTN causes different health-related problems on people beyond annoyance [2,6], such as cardiovascular diseases [7], hypertension [8], diabetes [9], among others. In order to address this problem in a harmonized manner, the European competent authorities defined and published the European Noise Directive 2002/49/EC (END) [10] in 2002, together with the subsequent Common Noise Assessment Methods in Europe (CNOSSOS-EU) [11,12]. Regarding these regulations, the European member states are asked to tailor strategic noise maps for large agglomerations (with more than 100,000 inhabitants) and key infrastructures (major roads, railways and airports) [10]. In addition, they have to inform citizens about their exposure to noise levels—typically differentiating day and night periods [10], besides defining and developing the corresponding action plans to mitigate the noise levels every five years where necessary.

These strategic noise maps have been hitherto developed from representative acoustic data collected by experts using certified devices, taking into account the urban spatial characteristics of their location for the proper simulation of sound propagation [13,14,15], among which the canyon effect in narrow streets becomes an important parameter to consider [16,17]. These data are subsequently fed into a precomputed acoustic model [18], after the manual removal of undesired acoustic events (e.g., sirens, airplanes, trams, etc.) to avoid biasing the noise map generation process [19]. Nevertheless, recent technological advances have enabled the development of alternative approaches within the Smart City paradigm, which encompasses a more interactive and responsive administration of cities to protect and improve the management of public spaces [20]. Hence, it leads to the development of new strategies to assess environmental noise in a more dynamic way than current approaches based on static noise maps by means of smart noise monitoring solutions. The combination of the Internet of Things (IoT) paradigm with the design and development of low-cost acoustic sensors has given rise to the so-called Wireless Acoustic Sensor Networks (WASNs) (the reader is referred to [21] for a review of the state-of-the-art of this topic). Recently, the WASN concept has been improved thanks to IoT-based advances, which have led to the miniaturization of the sensor electronics and the improvement of their lifetime [22], besides allowing the collection of representative acoustic data from the environment of interest. Several research projects have already deployed WASN-based dynamic noise monitoring systems. The first generation of WASN-based approaches have been mainly focused on measuring the global equivalent sound levels of the monitored locations [21]. Nevertheless, it is worth mentioning that some preliminary attempts have also been developed to identify specific acoustic events through a WASN. For instance, in the Sounds of New York City project (SONYC), the WASN-based system has been designed to analyse the distribution of the outdoor noise complaints and identify their origin [23]. However, the designed machine listening approach bases on an acoustic database that combines real-life samples with synthetically mixed audio excerpts, hindering the analysis of the natural characteristics of the sensed acoustic urban environments. Moreover, the LIFE DYNAMAP project has developed a WASN-based dynamic noise mapping system that includes sound event detection [24]. The project focuses on determining the acoustic impact of road infrastructures through the real-time monitoring of RTN levels solely (either from short or long distance traffic). To that effect, those events unrelated to regular road traffic are removed automatically to avoid biasing the computation of the A-weighted equivalent RTN levels ($L_{Aeq}$, in dBAs) through an Anomalous Noise Event Detector (ANED) [25]. According to the project specifications, those acoustic events that do not come from vehicles’ engines or from the contact of their tires with the road are denoted as Anomalous Noise Events (ANEs) [24]. ANEs were preliminary described through an expert-based recording campaign conducted before deploying the WASNs in the two pilot areas of the project: the District 9 of Milan to test the system across an urban area [26], and the A90 motorway that surrounds Rome to evaluate its performance on a suburban environment [27]. The conducted overall analyses showed the high diversity and occasional nature of this kind of events, which makes their modelling through synthetic audio mixtures almost unfeasible (see [28] for further considerations). In what concerns the urban environment, that study was somehow partial since the recording campaign was mainly focused on collecting ANEs through non-sequential diurnal recording periods, hindering their subsequent longitudinal analysis. Moreover, the samples were taken at the street level instead of the building façades, where the low-cost sensors have been finally placed.

With the aim of improving the characterization of urban ANEs in real acoustic environments in terms of their temporal and local particularities, this work analyses their distribution between day and night in narrow and wide streets through a WASN in real operation, considering the diurnal period as 06:00–22:00 [29] (based on the END recommendations [10]), and 1-lane roads as narrow streets; consequently, the nocturnal period is 22:00 to 06:00, and those roads with more than 1 lane are considered as wide streets.

Section 2 reviews relevant works about temporal and location-based acoustic measurements and audio databases obtained from real-life data or through WASNs in real-operation. Section 3 describes the WASN-based analysis methodology of urban ANEs during day and night in narrow and wide streets, including the creation of a labelled WASN-based acoustic database. Next, Section 4 presents the conducted experiments and the obtained results from the analysis of the collected urban ANEs. Finally, in Section 5, several relevant aspects regarding the conducted research are discussed, before presenting the main conclusions together with the future research lines.

## 2. Related Work

This section reviews relevant works about real-operation environmental acoustic databases and measurements. In the literature, several audio databases related to the development of machine listening algorithms have been designed for bench-marking purposes, being mainly oriented to the training and the evaluation of acoustic event detection and classification algorithms. Picaut [22] details in their recent paper that the miniaturization of the sensor electronic components and the accessibility of low-cost computing processors together with the improved performance of batteries, have increased the application of low-cost WASNs, widening the possibility of implementing this kind of networks as they can be composed of a larger set of nodes to collect more information (both raw acoustic data and equivalent sound level measurements).

### 2.1. Temporal and Location-Based Acoustic Measurements

In urban acoustic environments, time dependence of the measurements is a key issue to consider, especially for those conducted so as to build dynamic acoustic noise maps. The literature presents several studies around the temporal evolution of acoustic measurements in cities, mainly focused on road traffic noise. The first steps in dynamic noise mapping took into account which acoustic descriptors were better suited to capture urban traffic noise dynamics [30]. In this sense, the most relevant feature for assessing these dynamics appears to be traffic signal cycle, which corresponds to a certain temporal pattern evolution.

Moreover, it has been observed that in order to characterize environmental noise in situ by means of $L_{Aeq}$ measurements, mainly two periods of time should bee considered [10,29]: diurnal reference time ($L_{day}$) or nocturnal reference time ($L_{night}$), using a pre-set window with a closed observation period. The main goal is to determine the minimum measurement time interval to obtain an accurate estimation of $L_{Aeq}$ values, improving the stability of the measurements [31]. This is crucial when these values are used to update a dynamic noise map, which combines real-time measurements and data processing to assess the acoustic impact of noise sources. Obtaining the dynamics of the acoustic measurements across a city requires the application of a statistical approach, evaluating the noise trends at different streets [32], in order to draw the patterns of the $L_{Aeq}$ values for each location and time period properly. In [33], Lan presents a spatio-temporal noise distribution analysis with the aim of controlling and managing RTN by means of representative maps obtained for several periods of time. The approach assumes two variables that are time-dependent of traffic flow—traffic density and traffic speed—and evaluates the spatio-temporal characteristics of multi-source data to generate the corresponding RTN map. Finally, the paper also evaluates the noise distributions associated with different periods of time using the raw recorded audio.

Therefore, besides the temporal factor, the spatial characteristics of the location where the measurements are obtained, are also relevant for the correct analysis of the measurements. Several approaches have been considered to classify the different noise types and/or noise levels collected at specific urban sites. In [13], a categorization method for RTN evaluation is defined, assuming that urban noise is stratified according to five predefined categories based on mobility routes, which results to be appropriate for a city of nearly 320,000 citizens. The noise level predictive capacity of the method reached around 80%, which lead the authors to conclude that it could be a simple and low-cost method to conduct a statistical evaluation of the traffic noise in similar cities. A dynamic noise map obtained using more than 17 monitoring stations is described in [34] for the city of Badajoz, Spain. The noise sources considered are road and rail traffic, leisure, commercial and pedestrian derived noise. RTN is modelled independently from the other noise sources; an exhaustive road categorization was conducted considering all the streets network. The other noise sources (leisure, commercial and pedestrian zones) were characterized on the basis of noise measurements—gathered during more than one year—and taking into account more knowledge about the surroundings of the location to help their classification in terms of complex sources into the different street types.

Furthermore, the specific geometry of the streets is a key issue for the proper modelling of sound propagation. A systematic comparison between the sound fields in narrow street canyons with diffusely and geometrically reflecting boundaries is developed in [17] by means of the development of a radiosity-based theoretical model. The results obtained are substantially different depending on the chosen boundaries, recommending their design as diffusely reflective rather than acoustically smooth for better sound attenuation. The street canyon effect on sound pressure level (SPL) distribution is numerically studied with the full-wave finite-difference time-domain method in [16]. The study concludes that the building shape (up to 7 dBA), the building façade design (up to 12.9 dBA), the street geometry (up to 11.3 dBA) and the presence of furniture and other elements in the street can have a relevant impact on people’s noise exposure. Acoustic performance-based design is used in [35] to investigate the SPL reduction provided by the shape and the acoustic cladding of urban façades in front of a noise source in a street canyon. The results show that the works over the façade and overall, on the street paving, have a relevant impact on indoor noise propagation in buildings. For more references about the sound propagation in urban canyons, the reader is referred to [36], where several comparisons over simulated and measured SPLs on narrow urban canyons of the city of Vienna are described. The results show that the simulated and the measured values do generally agree, and that the developed simulator can be used for the planning of similar urban areas. Finally, the effect of urban spatial shape on sound propagation is evaluated in [15], going farther than theoretical spatial models. The proposal is based on considering high-density streets as items under study, and by means of reliable spatial parameters, the authors obtain the acoustic propagation data through computer simulations. Several metrics are evaluated, such as attenuation, reverberation and decay time depending on different spatial parameters, concluding that the sound propagation in urban streets is consistent with the characteristics of semi-free sound field propagation.

Finally, it is to note that the aforementioned works are mainly focused on RTN, paying no attention to the possible complex composition of the analysed acoustic environment, where the presence of other acoustic events such as ANEs, although occasional, can bias the acoustic measurements significantly [37,38].

### 2.2. Real-Life Audio Databases

The environmental acoustic databases described in the literature are usually employed by the machine listening research community to train and test different type of algorithms. They are generally composed of artificially generated sound mixtures or from real-life recordings. The former allows the control of the signal-to-noise ratio (SNR) of the synthetic audio mixtures [28], and dealing with class imbalance (i.e., data scarcity of specific audio categories) by means of data augmentation [39,40]. Nevertheless, although data augmentation techniques are very effective they hinder the analysis of the actual characteristics of real acoustic environments, which can only be conducted from real-life data.

Valero, in [41], presents an automatic approach for the classification of road vehicles based on their pass-by signature. The team recorded a dataset with six categories (light vehicles, heavy vehicles, motorcycles, aircrafts, trains and industrial noise), resulting in 90 real-life samples for each category, with a duration of 4 seconds each. Heittola, in [42], published a 1133 min audio database that includes 10 different acoustic environments from both indoor and outdoor recordings. Their goal is to detail how context information can be used for sound event detection; the approach pretends to simulate the human behaviour by means of a two-stage process that includes automatic context recognition and sound event detection, once the context has been identified. Despite the temporal component is taken into account, it does not refer to the dynamics or the location of the target sound, but to the surrounding events to improve its classification. Foggia, in [43], presents a large database of audio events in the framework of a surveillance application. The training dataset is about 20 h, while the test set is about 9 hours. In this work, the goal of the dataset generation is completeness in terms of events (short and long sounds) coexisting with diverse background noise levels. Moreover, the same research laboratory developed a smaller database of about 1 h duration also for surveillance purposes focused on road acoustic events, which contains sound events from tire skidding and car crashes [44].

Alías, in [28], presented a real-life acoustic database of 9 h and 8 min collected from the urban and suburban pilot areas of the LIFE DYNAMAP project [24] by means of expert-based recordings during non-sequential diurnal time periods mainly. This acoustic database was developed for discriminating ANEs from RTN through a sound event detection algorithm named ANED running on the low-cost acoustic sensors [25,45]. The ANEs, which correspond to the 7.5% of the labelled data, were classified into 19 different subcategories after expert annotation, and their SNRs were computed with respect to the background noise levels. The obtained SNRs ranged from −10 dB to +15 dB, showing also a wide heterogeneity of intermediate values. It is worth mentioning that the recordings in the urban area were collected at the street level in preselected locations across District 9 of Milan [32], while the recordings in the suburban area were conducted on several portals of the A90 ring-road surrounding Rome (see [27] for further details). In the final stage of the DYNAMAP project, the same authors have presented in [46] the production and overall analysis of a WASN-based environmental acoustic database collected through the 19-node WASN of the suburban area of Rome in real-operation. As a result, 156 h and 20 min of labelled audio data have been obtained, containing 16 ANE subcategories that correspond to 1.8% of the data, in contrast to the preliminary suburban expert-based dataset that contained 3.2% of ANEs throughout the total recorded time. A possible explanation to this difference is that the expert-based dataset recording was centred in day-time while the WASN-based database included also nocturnal samples, which showed a lower presence of ANEs. A complementary analysis to these works can be found in [38], which was focused on evaluating the aggregate impact of the ANEs occurring in the acoustic environments on the computation of $L_{Aeq}$ values. Nevertheless, none of these previous works developed within the DYNAMAP project pretended to study the temporal evolution of the characteristics describing ANEs at specific locations as they were analysed in an aggregate manner.

Another WASN-based project that has collected real-operation acoustic samples is SONYC [23]. The researchers provide a taxonomy of the urban sounds by means of a two-level hierarchy, dividing them into 8 coarse categories and 23 fine subcategories [47]. The generated database is composed of 2351 recordings in the train split and 443 in the validation counterpart, making a total of 2794 audios of 10-s each. Both the taxonomy and the details of the SONYC project database can be found in [48]. The most innovative proposal of this WASN-based approach is that by means of the deployed network, the distribution of the outdoor noise complains can be located, besides identifying whether they have been produced or not, e.g., due to an after-hour construction noise [23]. This process can be done by identifying the time of occurrence of the group of annoying events, allowing also the retrieval and visualization of the data streams obtained for each localized complain. Nevertheless, as the approach is based on Deep Learning models, it requires a huge amount of labelled data to work properly. To this aim, the considered acoustic model is trained by means of both real-life samples and artificially mixed audio excerpts through data augmentation techniques [23]; an approach that overrides the the use of the created database to analyse the actual characteristics of that urban acoustic environment.

Mesaros, in [49], describes an acoustic database recorded in multiple cities in Europe—which is an extension of the TUT 2018 Urban Acoustic Scenes dataset [50]. It contains recordings from Barcelona, Helsinki, London, Paris, Stockholm and Vienna, adding in the subsequent TAU 2019 dataset, Lisbon, Amsterdam, Lyon, Madrid, Milan and Prague. The recordings were conducted using four devices simultaneously: (i) Soundman OKM II Klassik/studio A3 electret binaural microphone, (ii) Samsung Galaxy S7, (iii) iPhone SE and (iv) GoPro Hero5 Session. Taking into account this variety of recording devices, the scenes were manually labelled to enable the training and test of the subsequently developed machine listening algorithms. The dataset was used in one of the DCASE 2019 Challenges that included data from different recorded acoustic scenes, using the acoustic raw pieces of audio together despite their different locations and origins.

## 3. WASN-Based Day–Night Analysis of ANEs in Narrow and Wide Streets

This section describes the development of an acoustic environmental database through a WASN in real operation (detailing the recording campaign methodology and the subsequent expert-based labelling process), together with the analysis methodology followed to characterize the distribution of ANEs between day (D) and night (N) time periods (06:00–22:00 and 22:00–06:00, respectively) in narrow (R) and wide (W) streets (1-lane and more than 1-lane roads, respectively), and their combination.

### 3.1. WASN-Based Recording Campaign and Expert-Based Labelling Process

**Figure 1:**
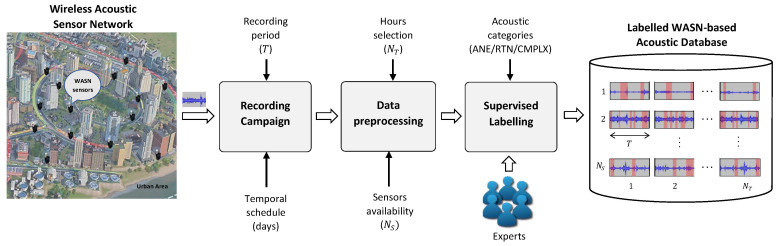
Block diagram of the generation process to develop a labelled Wireless Acoustic Sensor Network (WASN)-based acoustic database, where the labels are Anomalous Noise Event (ANE), Road Traffic Noise (RTN) and complex (CMPLX), denoting the latter complex audio passages.

In order to analyse the main characteristics of urban ANEs properly, it is necessary to collect a representative set of this kind of acoustic events in their natural state. Figure 1 shows the process to obtain a labelled WASN-based acoustic database through an acoustic sensors network in real operation. First, a WASN-based recording campaign has to be designed and performed across the area of interest, determining a recording period (*T*) together with a specific temporal schedule, e.g., specific recording hours during weekdays and/or weekend days. Once the recordings have been finished, the gathered raw acoustic data have to be preprocesed (i.e., cleaned, organized and selected, if necessary), considering the performance of the sensors—a relevant issue when collecting data through a WASN in real-operation since some sensors may present operational problems during the recording campaign. As a result, the $N_{S}$ sensors that captured enough representative data are selected, besides determining the corresponding set of $N_{T}$ time periods considered for the subsequent analyses.

Next, the preprocessed data are manually labelled by experts in acoustics and audio signal processing, considering visual information (e.g., waveform and spectrogram in dB) while listening to the recorded acoustic signals to annotate the events they contain. After showing the experts several representative examples, they are asked to classify each portion of audio according to the following criteria: (i) the audio excerpts that contain all kinds of sounds coming from vehicles’ engines and tires (even if they are distant or quite similar to background noise) should be labelled as RTN; (ii) the audio clips containing sounds unrelated to regular RTN should be labelled as ANEs, specifying their typology through a subcategory label defined and agreed during the labelling process; and finally, (iii) those audio passages difficult to classify as one of the aforementioned categories (e.g., containing a high diversity of sound sources with an origin hard to identify), should be labelled as complex (CMPLX) audio excerpts.

### 3.2. ANE Features Analysis Methodology

This section describes the methodology followed to analyse the main characteristics of the ANEs collected through a WASN in real operation for different acoustic environments, based on the extraction of several representative features, typically related to their presence and individual characteristics, such as event duration and SNR with respect to background RTN. In particular, the analysis focuses on the study of the particularities of these features for each ANE subcategory for day-and night-time periods plus narrow and wide street types.

**Figure 2:**
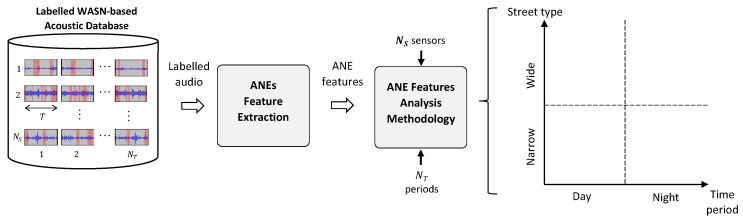
ANE feature analysis methodology from a labelled WASN-based acoustic database.

As can be observed from Figure 2, the analysis starts with ANEs feature extraction from the labelled acoustic database (see Figure 1). The analysis evaluates the particularities of the features considered to characterize the events composing the $N_{SC}$ ANEs subcategories for each subcategory *i*, period of time *j* (day or night) and type of street *k* (narrow or wide), regarding the number of recording periods $N_{T}$ ($N_{T}^{D}$ and $N_{T}^{N}$ for the diurnal and nocturnal periods, respectively), and/or number of streets $N_{L}$ ($N_{L}^{R}$ and $N_{L}^{W}$ for the narrow and wide streets, respectively). In particular, the day–night-based analysis is conducted through the aggregation of the ANE features along the corresponding $N_{R}^{L}$ and $N_{W}^{L}$ locations, with the aim of characterizing its temporal evolution disregarding the specific location where they were collected. Likewise, the considered features can be temporally aggregated for $N_{T}^{D}$ and $N_{T}^{N}$ to study their local particularities, evaluating the specific characteristics of the ANE subcategories for each group of narrow and wide streets. Finally, the analysis methodology also allows the study of the combination of day–night and narrow–wide street pairs.

### 3.3. Day–Night Plus Narrow–Wide Analysis

In order to analyse the features extracted from the ANE subcategories considering when and where they are collected, a day–night plus narrow–wide analysis is conducted. The approach followed to implement each one of these analyses is described in the following paragraphs.

- Day–Night analysis: With the aim of analysing the temporal evolution of ANE subcategories during day and night periods, a $P_{\mathrm{DN}}$ matrix (composed of $P_{D}$ and $P_{N}$ vectors) is computed for each extracted feature per ANE subcategory, disregarding their specific localization of observation, being defined as
  (1) $$P_{\mathrm{DN}}=\left(P_{D}^{\prime },P_{N}^{\prime }\right)$$
  (2) $$P_{D}=\left(p_{1}^{D},p_{2}^{D},\ldots ,p_{i}^{D},\ldots ,p_{N_{SC}}^{D}\right)$$
  (3) $$P_{N}=\left(p_{1}^{N},p_{2}^{N},\ldots ,p_{i}^{N},\ldots ,p_{N_{SC}}^{N}\right)$$
  where operator ${}^{\prime }$ represents vector transposition and $p_{i}^{j}$ stands for the result of the considered statistical measure (e.g., median, mean, maximum, minimum, total, etc.) computed for each feature considered to parameterizes the ANE subcategory *i* (for $i=\{1,2,\ldots ,N_{SC}\}$) within each time period *j* —diurnal or nocturnal—by taking into account the full set of $N_{S}$ sensors of the database. The street-based aggregation process depends on the considered statistical measure. For instance, if it represents a quantity, then the aggregate operation is a sum of the corresponding values obtained within the given time period for all streets. However, if the feature is characterized by a statistical parameter (e.g., median or mean values), then it should be recomputed considering the full set of values of the analysed feature for all the locations.
  Finally, it is to note that each vector value is averaged within the total number of hours contained in the $N_{T}^{D}$ and $N_{T}^{N}$ recording periods considered to sample the diurnal and nocturnal periods, respectively.
- Narrow–Wide analysis: The local particularities of ANE subcategories observed between narrow and wide streets for a specific ANE feature are described by $P_{\mathrm{RW}}$ matrix, which is composed of $P_{R}$ and $P_{W}$ vectors of parameters computed throughout the day for each group of streets, being represented as
  (4) $$P_{\mathrm{RW}}=\left(P_{R}^{\prime },P_{W}^{\prime }\right)$$
  (5) $$P_{R}=\left(p_{1}^{R},p_{2}^{R},\ldots p_{i}^{R},\ldots ,p_{N_{SC}}^{R}\right)$$
  (6) $$P_{W}=\left(p_{1}^{W},p_{2}^{W},\ldots p_{i}^{W},\ldots ,p_{N_{SC}}^{W}\right)$$
  where operator ${}^{\prime }$ represents vector transposition and $p_{i}^{k}$ denotes the result of the considered statistical measure (e.g., median, mean, maximum, minimum, total, etc.) computed for each feature that parameterizes ANE subcategory *i* (with $i=\{1,2,\ldots ,N_{SC}\}$) collected throughout the day from each set of sensors *k*—located at narrow streets and wide streets, respectively. As for the day–night analysis counterpart, the narrow–wide aggregation process depends on the selected statistical measure. For instance, if it is parameterized quantitatively, the aggregate value is obtained by simply summing all the values within the given subset of streets for all the time periods. On the other hand, if the feature is statistically parameterized (e.g., through median or mean values), the statistical parameter is computed encompassing the full set of values for all the recording time periods within each group of streets. Moreover, all vector values are averaged considering the number of sensors’ locations $N_{L}^{R}$ and $N_{L}^{W}$ placed in narrow and wide streets, respectively.
- Day–Night plus Narrow–Wide analysis: This analysis is based on considering the day–night and narrow–wide pairs combination to study the particularities of a specific ANE feature computed from the labelled ANE subcategories for all the day–night periods plus group of streets, obtaining the following matrix and vectors of parameters for each pair of possible combinations
  (7) $$P_{\mathrm{DN}}^{\mathrm{RW}}=\left(P_{RD}^{\prime },P_{RN}^{\prime },P_{WD}^{\prime },P_{WN}^{\prime }\right)$$
  (8) $$P_{DR}=\left(p_{1}^{DR},p_{2}^{DR},\ldots p_{i}^{DR},\ldots ,p_{N_{SC}}^{DR}\right)$$
  (9) $$P_{NR}=\left(p_{1}^{NR},p_{2}^{NR},\ldots p_{i}^{NR},\ldots ,p_{N_{SC}}^{NR}\right)$$
  (10) $$P_{DW}=\left(p_{1}^{DW},p_{2}^{DW},\ldots p_{i}^{DW},\ldots ,p_{N_{SC}}^{DW}\right)$$
  (11) $$P_{NW}=\left(p_{1}^{NW},p_{2}^{NW},\ldots p_{i}^{NW},\ldots ,p_{N_{SC}}^{NW}\right)$$
  where operator ${}^{\prime }$ represents vector transposition and $p_{i}^{jk}$ denotes the result of the considered statistical measure (e.g., median, mean, maximum, minimum, number of, etc.) computed for each feature that parameterizes the ANE subcategory *i* (for $i=\{1,2,\ldots ,N_{SC}\}$) during the *j*-th time period—diurnal or nocturnal—within the *k*-th group of streets—narrow or wide—by considering the corresponding recording time periods $N_{T}^{jk}$ of each jk pair combination. Moreover, all vector values are averaged on the total recorded time from the diurnal and nocturnal periods (i.e., $T\times N_{T}^{D}$ and $T\times N_{T}^{N}$), respectively, as well as the number of locations placed in narrow and wide streets, denoted as $N_{L}^{R}$ and $N_{L}^{W}$, respectively.

## 4. Experiments and Results

This section describes the process followed to generate the urban WASN-based acoustic database developed using the low-cost acoustic sensors installed across the urban pilot area of the DYNAMAP project, together with its main features. Moreover, it details the conducted experiments and the results obtained from the study of the main characteristics of the collected urban ANE subcategories following the described analysis methodology.

### 4.1. Development of the WASN-Based Urban Database

**Figure 3:**
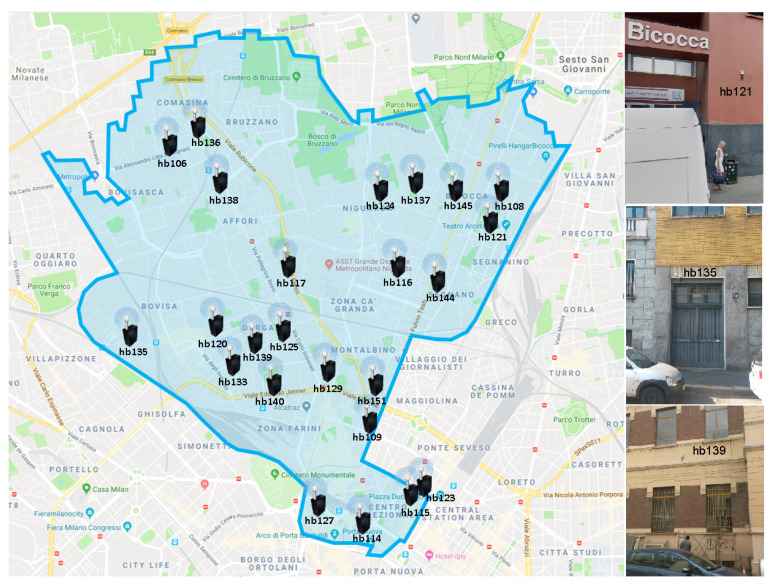
Map of the location of the 24-nodes of the WASN deployed within the DYNAMAP’s urban pilot area across the District 9 of Milan (**left**), showing the exact installation of three of them in the building facades (**right**) for illustrative purposes.

This section details the process followed to obtain the labelled WASN-based database from the different acoustic environments monitored across the District 9 of Milan. Figure 3 shows the location of the 24 low-cost sensors of the WASN deployed in that urban area together with their Ids—the details of their specific location are detailed in Table 1, together with three pictures showing the exact sensor installation in the building façades for illustrative purposes. It is worth mentioning that the sensors were designed to measure $L_{Aeq,1s}$ (i.e., $L_{Aeq}$ computed every second) and to evaluate whether each measurement corresponds to RTN or ANE by running the ANED algorithm, besides allowing occasional audio recordings accessible through 3G connection [51].

In what concerns the design of the WASN-based recording campaign (first block of Figure 1), two days of the same week during autumn 2017 were sensed through the network nodes to sample RTN and anomalous noise events in different traffic conditions between a weekday (Tuesday, November 28) and weekend day (Saturday, December 2). The acoustic data were recorded in continuous audio clips (with 48 kHz sampling frequency), retrieving the first T=20 min of each sampled hour × 24 h/day, considering nodes’ storage capacity limitations. Following a similar approach as the one considered for the generation of the suburban WASN-based database [46], during the data preprocessing stage (second block of Figure 1) up to $N_{T}=11$ periods were selected for the weekday (i.e., 02:00, 03:00, 05:00, 08:00, 09:00, 11:00, 14:00, 15:00, 17:00, 20:00 and 23:00), and $N_{T}=9$ for the weekend day (i.e., 02:00, 05:00, 08:00, 11:00, 14:00, 17:00, 20:00, 21:00 and 23:00), as a trade-off between data representativeness and annotation effort to sample the diversity of each acoustic environment (e.g., the traffic flow variability is higher during the day than at night).

As a result of the WASN-based recording campaign, 154 h and 20 min of environmental audio data were collected. However, after analysing the availability sensors, it was observed that 4 out of the 24 nodes presented some kind of difficulty in the recording process due to diverse operational problems. Among them, it is worth mentioning that sensor hb114 registered only 7 periods of time on the weekend day (at 03:00, 05:00, 06:00, 08:00, 11:00, 15:00 and 18:00), while three other sensors missed one or two recording periods (specifically, hb116 missed 02:00 and 03:00 on the weekend, hb117 23:00 on the weekend and hb138 15:00 and 23:00 on the weekday and 14:00 on the weekend). As a consequence, after data cleaning and selection, only recordings from $N_{S}=23$ sensors were taken into consideration for the subsequent analyses, discarding the data obtained from sensor hb114 due to their lack of representativeness. Next, the selected recordings were organized in separated raw WAV audio files (one for each audio clip of T=20 min), and labelled with the corresponding sensor Id, day and starting time of the recording. As a result, a total of 453 files were considered for the subsequent analyses, encompassing 151 h of environmental audio data.

Finally, the WASN-based acoustic audio files were manually labelled by 5 experts in audio signal processing using the Audacity software to perform the labelling process with the aid of spectrograms while listening to the recorded signals (last block of Figure 1). As a result, each piece of acoustic data was assigned to RTN, COMPLX or ANE categories, being the latter subcategorized into 26 urban-like sounds (see Table 2). Concretely, up to 126 h and 43 s were classified as RTN (83.5%), 13 h 8 min and 48 s were labelled as one of the 26 ANEs subcategories (8.7%) and the remaining audio passages (11 h 50 min and 29 s) were tagged as CMPLX (7.8%), a category which analysis is left for future investigations.

### 4.2. ANEs Feature Extraction and Parameterisation

The first step to analyse the characteristics of the collected anomalous noise events is feature extraction. To that effect, the ANE subcategories are parameterized based on their presence, together with distinctive characteristics related to their duration and contextual SNR distributions.

- Occurrence: The presence of each ANE subcategory is described by two statistic parameters: the total number of times it is observed [28], and the mean number of times it is observed within day–night periods and/or narrow–wide streets.
- Duration: The duration of each event is computed (in s) as the difference between the start and end points of each event present in the database [28]. This feature is statistically parameterized with a two-fold aim. On the one hand, by the median of its distribution to analyse the individual duration of each ANE subcategory, and on the other hand, by accounting for the total time a specific ANE subcategory is present [46] within each day–night periods and/or narrow–wide streets, besides computing the mean total duration of the events with respect to the corresponding time period and/or group of streets.
- SNR: The signal-to-noise ratio (in dB) of each event is computed as the ratio between the power of the ANE and the power of the surrounding RTN, following the classical SNR computation, so as to represent the inherent median acoustic salience of the event for a given set of streets and/or time period. To this aim, each ANE is considered the informative signal, being its surrounding RTN the noise signal, computing its SNR as [52]
  (12) $$SNR=10\;log_{10}\left(\frac{P_{ANE}}{P_{RTN}}\right),$$
  where
  (13) $$P_{x}=\sum_{n=1}^{N_{x}}\left(\frac{x^{2}\left[n\right]}{N_{x}}\right),$$
  and x[n] stands for the audio signal vector of $N_{x}$ samples length for either the ANE or the RTN signals of interest.
  Following the same approach considered for the duration, this variable is also statistically parameterized by the median value of each ANE subcategory distribution calculated under the premises of the analysis methodology detailed in Section 3.

### 4.3. Overall Analysis

In order to have a global picture of the sensed urban acoustic environments, this section describes the results of the overall analyses conducted on the audio excerpts labelled by the experts as an ANE subcategory, according to their presence (in number of occurrences), duration (in s) and SNR with respect to the background traffic noise (in dB).

As aforementioned, 26 ANE subcategories related to urban-like sound events were identified during the expert-based labelling process. It is worth mentioning that two of them were only observed during the working day, specifically, *rain* and *thun*, which are derived from a stormy episode. As can be observed from Table 2, those ANE subcategories with the largest number of occurrences are mainly events of short nature like *peop* (5822 occurrences, which account for 22.0% of total ANE occurrences), *bird* (4215 occurrences, 16.0%), *door* (3843 occurrences, 14.5%), *step* (3574 occurrences, 13.5%) and *brak* (3245 occurrences, 12.3%). Moreover, ANE subcategories with moderate number of occurrences are *wrks* (1045 occurrences, 4.0%), *horn* (957 occurrences, 3.6%), *bike* (943 occurrences, 3.6%), *dog* (649 occurrences, 2.5%), *bell* (311 occurrences, 1.2%) and *busd* (277 occurrences, 1.0%). The remaining ANE subcategories present few instances (less than 1%), being *wind*, *blin* and *tran*, which are rarely found in the recordings (less than 0.05%), and, finally, *thun*, which was only observed once, due to the fact that the ANEs related to weather events (*thun* and *rain*) were only observed during the weekday as aforementioned.

In what concerns the total duration of ANE subcategories, it can be observed from Table 2 that *peop* is the most active subcategory (9452.7 s), followed by two ANEs derived from traffic, in particular, *brak* (5054.9 s) and *sire* (3980.3 s), among which we also find street works (i.e., *wrks* with 4222.9 s of total duration). Moreover, the presence of *bird* and *airp* subcategories are also common in the urban environment, with an accumulated duration of 3632.3 s and 3441.7 s, respectively. Finally, it is also worth noting the relevant duration of the *inte* subcategory derived from industry (2703.6 s), due to its high median duration despite its modest presence, together with events of burst-like nature like *step* and *door* with 2162.8 s and 2096.7 s, respectively, thanks to their common presence in the urban environment. The remaining ANE subcategories represent less than the 4% of the accumulated total duration of the events.

Moreover, the global statistics of the individual particularities of the ANE subcategories in terms of their median duration and SNR are also shown in Table 2. It can be observed that the subcategory that presents the largest median duration is *inte* with a median length of 20.5 s. Next, *sire* and *airp* show median lengths of 9.67 and 9.3 s, respectively, which are followed by events derived from means of transportation such as *tran*, *tram* and *rubb*, together with *rain*, presenting a median duration between 6 and 8 seconds. Furthermore, *blin*, *alrm*, *trll*, *wind*, *musi*, *wrks*, *bell* and *brak* represent a quite diverse set of shorter events with median durations between 1 and 3 s. Finally, the remaining ANE subcategories reveal a burst-like nature with a median duration shorter than 1 s (e.g., see *horn*, *peop*, *glas*, *step*, *bird*, *dog*, among others).

Regarding the SNR distribution of the ANE subcategories, those events presenting the highest SNRs are *blin* and *dog* with median SNR values between 4 and 7 dB. These events have typically been found in audio passages with very low RTN levels. Moreover, the WASN-based recording campaign collected a weather-related event with high SNR (*thun* with a SNR of 5.7 dB). Furthermore, *tram* also presents quite high median SNR values, followed by *glas*, *bell*, *horn*, *tran*, *door*, *peop* and *rubb* with median SNR values between 1.5 dB and 3.5 dB. Finally, it is to note that several ANE subcategories such as *inte*, *rain* and *wind* show very low SNR values, being most of them below the power of the surrounding RTN. This can be explained by the restrictions of the method used for SNR computation that is based on estimating background noise level from the ANE neighbouring $L_{Aeq}$ levels (see [52] for further details), which may bias the result when the surrounding background noise or event is louder than the one under analysis, yielding to negative SNR values.

### 4.4. Day–Night Evolution of Urban ANEs

In this section, we analyse the temporal evolution of the collected urban ANE between day and night without taking into account their specific localization across the urban environment. To that end, matrices of parameters $P_{\mathrm{DN}}$ (see Equation (1)) are computed for each sensed day (weekday and weekend day), taking into account the four considered ANE features (occurrences, total duration and individual duration and SNR).

Table 3 shows the number of occurrences and total duration of all ANEs per diurnal and nocturnal periods. For a fair comparison, we compute the mean number of occurrences and total duration of the ANEs with respect to the total recording length for each time period in hours (i.e., 2.33 h of day-time and 1.33 h of night-time for weekday, plus 2 h of day-time and 1 h of night-time for weekend day). The table shows the higher presence of ANEs in diurnal periods with respect to the nocturnal periods for both week and weekend days. This pattern is slightly more noticeable in terms of mean total duration (125.5% greater for day than night) than for the mean number of occurrences (91.5% greater for diurnal periods) in weekday. However, during weekend the pattern is the reverse, being greater the difference of diurnal occurrences with respect to the nocturnal period (112.7% greater) than the mean total duration per hour (93.4% greater for day-time). On the other hand, there is also a significant increase in the number of events during the weekday compared to the weekend day, which is larger in terms of the mean total duration during day-time (39.5% greater) and for mean occurrences at night (34.2% greater) than at night for total duration (19.6% greater) and during day-time for the mean number of occurrences (20.8% greater).

**Figure 4:**
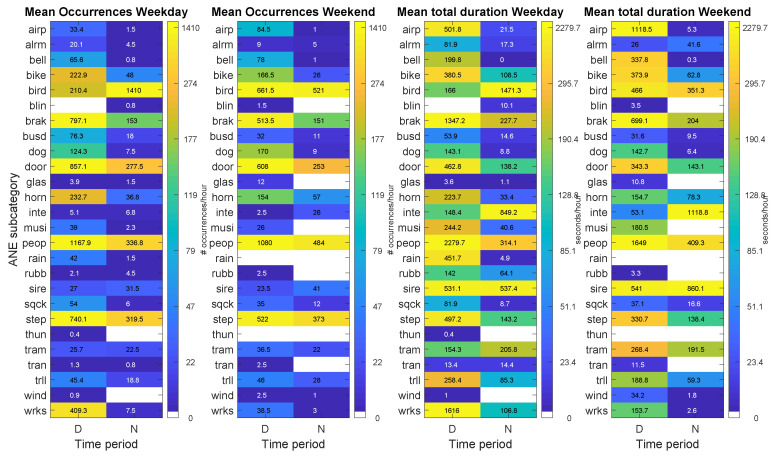
Results of the day–night analysis ($P_{\mathrm{DN}}$ matrices) differentiating weekday and weekend days for the following ANE features: mean number of occurrences per hour (two leftmost subfigures) and total duration per hour (two rightmost figures). Each subfigure shows the distribution of the corresponding ANE feature in terms of ANE subcategory and time period.

Figure 4 shows the results of the day–night analysis of the occurrences of ANE subcategories and their total duration during the weekday and the weekend day, respectively. First of all, the figure confirms the higher presence of anomalous noise events in the diurnal period. Specifically, those ANE subcategories with greater diurnal presence (more than 80% of their occurrences) are: *bell*, *bike*, *rain*, *wrks*, *wind*, *dog*, *airp*, *peop*, *musi* and *glas*. However, several ANE subcategories present a different behavior. On the one hand, *tram* and *sire* present a distribution of both occurrences and total durations quite similar between day and night-time periods. On the other hand, other ANE subcategories show a higher presence at nights, such as *inte* and *bird*. Moreover, it can be observed that ANE subcategories related to meteorological phenomena, like *rain* and *thun* are mainly present in the diurnal period of the weekday, and other events such as *blin*, *glas*, *tran*, *thun* and *wind* present a quite sparse distribution mainly due to the fact that these subcategories are the ones observed with the lowest number of occurrences and, thus, total durations (see Table 2). Finally, it worth noting that the differences of the mean number of occurrences between day and night are statistically significant for both the weekday ($\chi^{2}$ (24, N = 7927) = 2741.92, *p*<0.01), and weekend day ($\chi^{2}$ (21, N = 6339) = 419.69, *p*<0.01).

**Figure 5:**
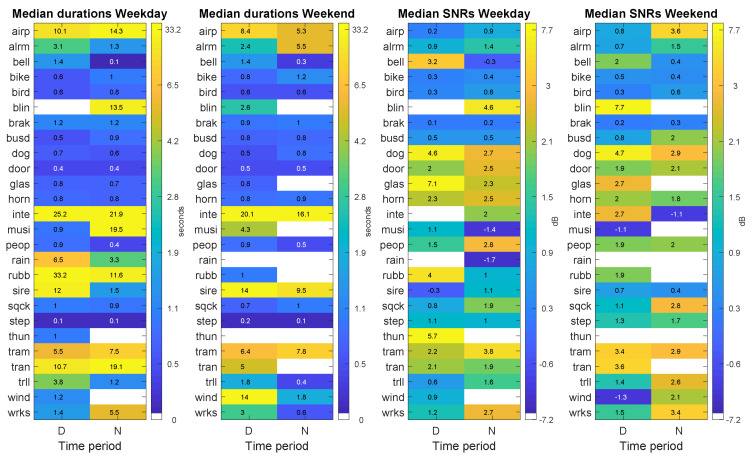
Results of the day–night analysis ($P_{\mathrm{DN}}$ matrices) differentiating weekday and weekend days for the following ANE features: median duration (two leftmost subfigures) and median SNR (two rightmost figures). Each subfigure shows the distribution of the corresponding ANE feature in terms of ANE subcategory and time period.

Figure 5 depicts the results of the day–night analysis of ANE subcategories in terms of their median duration and median SNRs during the weekday and the weekend day, respectively. It can be observed that the ANE subcategories that present higher median duration are *inte*, *rubb*, *tram*, *tran*, *airp*, *sire* and *rain*, while events such as *step*, *door*, *bird*, *dog*, *peop*, *horn*, *busd*, *glass*, *bell*, *bike*, *sqck* present very short median durations, since most of them entail a burst-like pattern. In terms of night vs. day variation, most ANE subcategories show stable patterns without significant variations. However, there are several subcategories that present quite relevant differences in terms of the variation of the median durations between day and night in both days (e.g., see *blin*, *musi*, *rubb*, *tran* and *wind*). Nevertheless, notice that these anomalous events do not present a homogeneous distribution throughout the day. For instance, *rubb* and *musi* are mostly observed on the weekday and during the diurnal and nocturnal periods, respectively. Regarding the statistical differences of the individual event durations between day and night for both days, they have been analysed using the Mann–Whitney U-test, concluding that those ANE subcategories that show significant differences (with p<0.01) are *bird*, *peop*, *sire* and *trll* on the weekday, and *peop*, *step* and *trll* on the weekend.

On the other hand, it is worth mentioning that most SNRs are higher at night for both sensed days, i.e., for 13 out of 21 ANE subcategories at weekday and for 12 out of 19 subcategories observed in both time periods. In particular, those ANE subcategories with higher nocturnal median SNR (with differences with respect to diurnal values greater or equal than 1 dB) are *inte*, *rain*, *sqck*, *tram*, *trll* and *wrks* on the weekday, and *airp*, *airp*, *busd*, *sqck*, *trll*, *wind* and *wrks* on the weekend day. These results can be explained due to the fact that RTN noise levels are typically lower at night, which makes ANEs become more salient. However, beyond this general pattern, it is to note that several ANE subcategories present quite significantly higher median SNR values during the diurnal period, such as *bell*, *dog*, *glas*, *musi* and *rubb* in weekday (between 1.9 and 4.8 dB higher) and *bell*, *dog* and *inte* for weekend (between 1.5 and 3.9 dB higher). Nevertheless, as aforementioned, some of the ANEs subcategories are particularly found in either day or night periods, a fact that could bias the qualitative analyses. Furthermore, when comparing median SNR values between weekday and weekend days, *glas*, *musi*, *rubb* and *wind* are those ANE subcategories entailing higher values for the weekday (between 2.1 and 5.8 dB higher), while *airp* and *tran* show higher SNR median values on the weekend (between 0.6 and 2.7 dB higher). The ANE subcategory *inte* presents a daily median SNR value 7.6 dB higher in the weekend than during the weekday, while this measure is 3.1 dB higher for the nocturnal period on the weekday than during the weekend. Finally, we have evaluated the differences of the individual SNRs of the ANE subcategories between day and night for both recording days through a Mann-Whitney U-test, concluding that those ANE subcategories that show significant differences (with p<0.01) are *bird*, *dog*, *door*, *peop*, *step* and *wrks* for the weekday and *bird*, *step* and *trll* for the weekend.

### 4.5. Particularities of Urban ANEs in Narrow and Wide Streets

In this section, we present the results of the analysis of the local particularities of the collected urban ANE subcategories in narrow and wide streets. To that end, $P_{\mathrm{RW}}$ matrices (see Equation (4)) are computed for each sensed day, taking into account the four considered ANE features (occurrences, total duration, median duration and median SNRs) by considering narrow streets those sensor locations placed at 1-lane rows and wide streets, for the rest (see Table 1).

Table 4 shows the mean number of occurrences and mean total duration of all ANEs analysed regarding the type of street where sensors are located. For a fair comparison, both mean values are averaged by the number of streets belonging to each type, that is, $N_{L}^{R}=11$ for narrow and $N_{L}^{W}=12$ for wide streets, respectively, after discarding the location of sensor hb114 for technical problems during the WASN-based recording campaign (see Section 4.1). As can be seen from the table, narrow streets tend to contain a larger presence of ANEs both in terms of number of occurrences (between 26% and 74% of increase with respect to wide streets), and total duration (between 58% and 89% of increment in narrow streets). In concordance to what has been observed in the day–night analysis (see Section 4.4), the weekday contains a higher averaged number of occurrences and total duration of ANEs.

**Figure 6:**
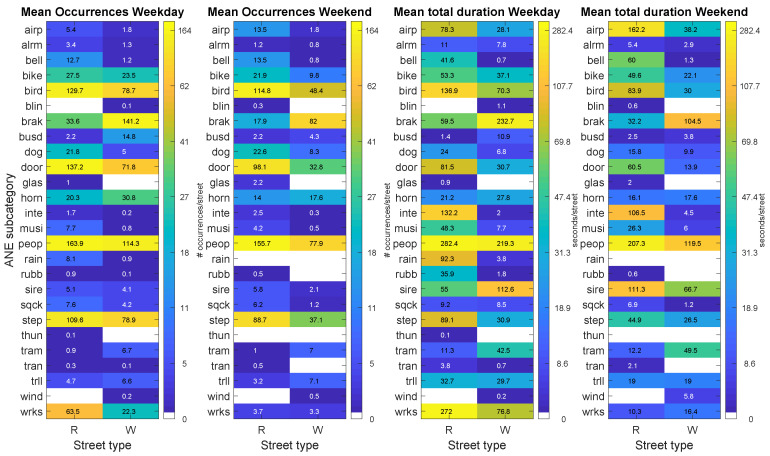
Results of the narrow–wide analysis ($P_{\mathrm{RW}}$ matrices) differentiating weekday and weekend days for the following ANE features: mean number of occurrences per street (two leftmost subfigures) and total duration per street (two rightmost figures). Each subfigure shows the distribution of the corresponding ANE feature in terms of ANE subcategory and type of street where the sensor is located.

Figure 6 shows the narrow–wide analysis matrices $P_{\mathrm{NW}}$ for the mean number of occurrences and total durations of ANE subcategories for both recording days. As can be observed, the following list of ANE subcategories appear to be more prominent in narrow than in wide streets: *peop* (between 56 and 65% of its occurrences), *step* (between 60 and 73% of its total durations), *bell* (more than 97% of its total durations), *inte* (more than 95% of its total durations), *musi* (more than 88% of its occurrences), *door* (more than 70% of its total durations), *wrks* (72.3% of occurrences during weekday), *sqck* (between 62 and 83% of occurrences), *airp* (more than 73% of occurrences), *tran* (more than 83% of total durations) and *rubb* (more than 94% of total durations). In contrast, several ANEs derived from means of transportation are more noticeable in wide streets, like vehicle brakes (*brak*, more than 82% of its occurrences), *busd* (more than 68% of its occurrences) or *tram* (about 88% of its occurrences). Finally, notice that the differences between the mean number of occurrences observed in narrow and wide streets were found statistically significant for the weekday ($\chi^{2}$ (21, N = 1379) = 172.83, *p*<0.01) and for the weekend day ($\chi^{2}$ (20, N = 936) = 142.39, *p*<0.01).

**Figure 7:**
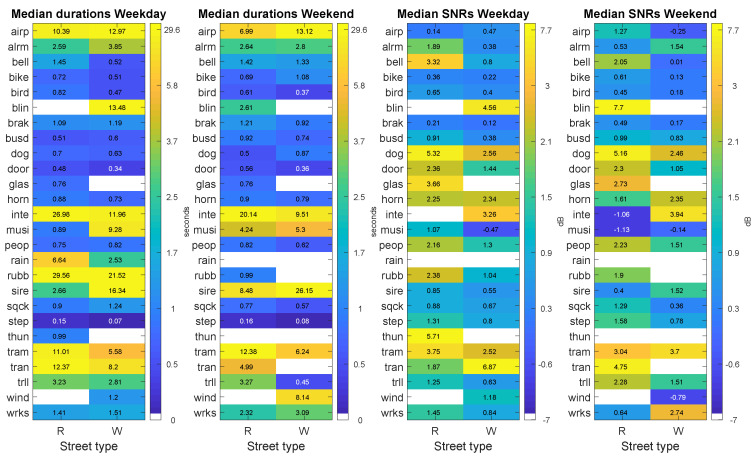
Results of the narrow–wide analysis ($P_{\mathrm{RW}}$ matrices) differentiating weekday and weekend days for the following ANE features: median duration (two leftmost subfigures) and median SNR (two rightmost figures). Each subfigure shows the distribution of the corresponding ANE feature in terms of ANE subcategory and type of street where the sensor is located.

Figure 7 shows the narrow–wide analysis matrices $P_{\mathrm{RW}}$ for the median duration and median SNRs of the ANE subcategories during the weekday and the weekend days, respectively. It can be observed that most ANE subcategories present quite similar median duration in both type of streets. However, it is worth mentioning that certain subcategories show some kind of imbalance between narrow and wide streets. The statistical analysis of the differences of the individual event durations based on the computation of the Mann–Whitney U-test confirm that there are several ANE subcategories that present significant differences (with p<0.01) between narrow and wide streets, such as *bell*, *bike*, *bird*, *door*, *horn*, *musi*, *sire*, *sqck*, *step* and *tram* for the weekday and *airp*, *bird*, *brak*, *dog*, *door*, *horn*, *peop*, *sire*, *step*, *tram* and *trll* for weekend day.

On the other hand, when analysing median SNRs, mostly narrow streets tend to present higher values, which are found in both sensed days for *bell*, *bike*, *bird*, *brak*, *busd*, *dog*, *door*, *peop*, *sqck*, *step* and *trll*. All of these ANE subcategories show SNRs between 0.09 and 2.76 dB higher in narrow streets compared to wide streets. Finally, only *inte* subcategory shows higher SNR values in wide streets than narrow streets for both days (between 5.00 and 6.94 dB higher), maybe due to the fact that the origin of this ANE is very local, as aforementioned. Nevertheless, this hypothesis should be confirmed through further analyses. In what concerns the statistical analyses of the differences between the individual SNRs of the events in narrow and wide streets, the Mann–Whitney U-tests conducted for each recording day proved that the following ANE subcategories show significant differences: *bell*, *bird*, *dog*, *door*, *peop*, *step* and *wrks* for the weekday and *bird*, *brak*, *dog*, *door*, *horn*, *peop* and *step* for the weekend.

### 4.6. Day–Night Plus Narrow–Wide Characteristics of Urban ANEs

In order to have a more detailed analysis of the characteristics of the collected ANE subcategories, this section describes the results of the day–night plus narrow–wide combinations based on the computation of $P_{\mathrm{DN}}^{\mathrm{RW}}$ matrices (see Equation (7)) for the considered features to parameterize the ANE subcategories and the two days under study. In this case, the mean values of the features are averaged considering $N_{L}^{R}=11$ narrow and $N_{L}^{W}=12$ wide streets and the total recorded time for each period, that is: 2.33 h of day-time (for DR and DW) and 1.33 h of night-time (for NR and NW) on the weekday, plus 2 h of day-time (for DR and DW) and 1 h of night-time (for NR and NW) during the weekend.

Table 5 shows the mean values of the number of occurrences and total durations for the day–night plus narrow–wide pairs. As can be observed, narrow streets reveal the highest presence of ANEs during day-time both in terms of occurrences and accumulated duration. When compared to the other three pairs, the mean number of occurrences per hour and street are 29.2–134,7% greater on the weekday and 64–324.5% greater on the weekend, while for the mean total duration this increase is between 58.3 and 261.3% during the weekday and between 79.1 and 302.5% on the weekend. Thus, in this analysis the dominant factor seems to be the time period in front of the street type because the second combination that entails a larger presence of ANEs is the DW pair. In this combination, the mean number of occurrences is 55.4–81.7% higher than the other two night-based pairs (NR and NW) for the weekday, and between 16–158.8% on the weekend, while the mean total duration is 41.3–128.3% larger for the weekday, and 122.9% larger than the NW configuration during the weekend.

**Figure 8:**
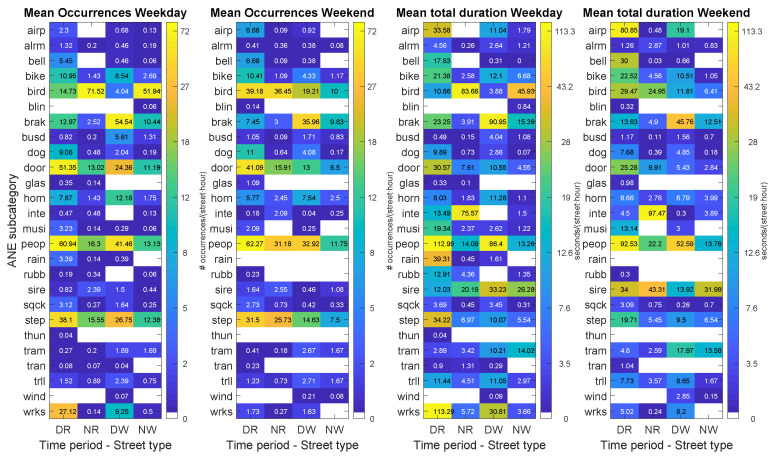
Results of the day–night and narrow–wide pairs analysis ($P_{\mathrm{DN}}^{\mathrm{RW}}$ matrices) differentiating weekday and weekend days for the following ANE features: mean number of occurrences per street and hour (two leftmost subfigures) and total duration per street and hour (two rightmost figures). Each subfigure shows the distribution of the corresponding ANE feature in terms of ANE subcategory and the pair street type and time period.

Figure 8 shows the results of the analysis of the mean number of ANE occurrences and total duration for both the weekday and weekend days for the four considered configurations. It can be observed that narrow streets host most of the occurrences during day-time (DR). More precisely, several ANE subcategories like *airp*, *bell*, *bike*, *dog*, *door*, *glas*, *musi*, *peop*, *sqck*, *wrks* and *thun* present in DR more than 45.1% of their total number occurrences and more than 37.3% of their total duration for both days. At second level, wide streets contain at least six sources of anomalous events during the diurnal period (DW) (i.e., *brak*, *busd*, *horn*, *tram*, *trll* and *wind*) with more than 33.4% of their occurrences and more than 33.6% of their total duration in DW for both days. Narrow streets (NR) contain more than 34.8% of their occurrences and 22% of their total duration at night for *bird*, *inte* and *sire* for both sensed days. Moreover, we want to highlight that the *tram* subcategory is the one that presents a quite important presence in wide streets at night (NW pair), with 33.8% of its occurrences and total duration. Finally, regarding the statistical comparison between the mean number of occurrences found among the four combination pairs, again they present significant differences for both the weekday ($\chi^{2}$ (54, N = 685) = 348.35, *p* < 0.01), and the weekend day ($\chi^{2}$ (51, N = 557) = 140.71, *p* < 0.01).

**Figure 9:**
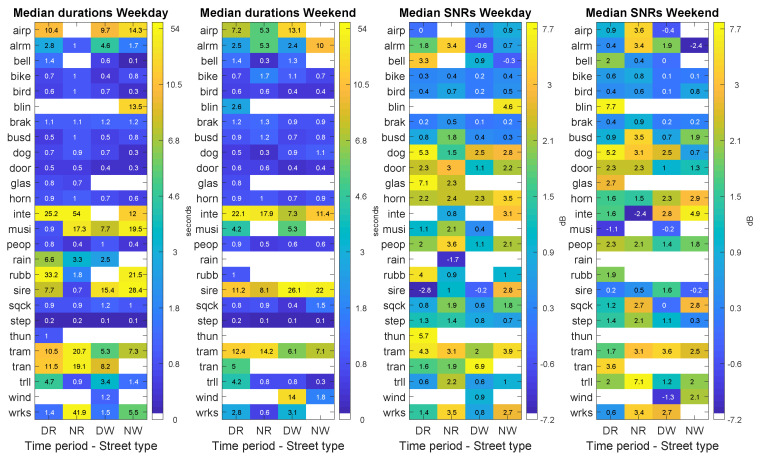
Results of the day–night and narrow–wide pairs analysis ($P_{\mathrm{DN}}^{\mathrm{RW}}$ matrices) differentiating weekday and weekend days for the following ANE features: median duration (two leftmost subfigures) and median SNR (two rightmost figures). Each subfigure shows the distribution of the corresponding ANE feature in terms of ANE subcategory and the pair street type and time period: Narrow–Day (RD), Narrow–Night (RN), Wide–Day (WD) and Wide–Night (WN).

Figure 9 depicts the results of the computation of the median durations and median SNRs of the anomalous events for both recording days. It can be observed that narrow streets present the highest number of events with maximum median duration, for 11 ANE subcategories at night on the weekday and during day-time on the weekend. Regarding the individual durations of the events, it can be observed that most events present quite stable patterns among the four combination pairs. However, several ANE subcategories present relevant variations. The conducted statistical analyses based on the Kruskall–Wallis test prove that the differences of individual event durations are significantly different (with *p* < 0.01) among the four configurations for the following ANE subcategories: *bell*, *bike*, *bird*, *door*, *horn*, *musi*, *peop*, *sire*, *step* and *tram* on the weekday, and *bird*, *brak*, *door*, *horn*, *peop*, *sqck*, *step*, *tram* and *trll* on the weekend.

In what concerns the analysis of median SNRs, the highest values are found at night on narrow streets, for 13 ANE subcategories on the weekday and 9 subcategories on the weekend. Within these subsets, the subcategories with highest median SNR values are *alrm* (3.4 dB), *busd* (between 1.8 and 3.5 dB), *door* (between 2.3 and 3 dB), *step* (between 1.4 and 2.1 dB), *trll* (between 2.2 and 7.1 dB) and *wrks* (between 3.4 and 3.5 dB). On a second level, narrow streets contain the next group of higher median SNRs during day-time, for 6 ANE subcategories on the weekday and 7 on the weekend day. Moreover, lower median SNR values are found in the diurnal period in wide streets, more concretely, 10 ANE subcategories present their minimum SNRs on the weekday, becoming 11 during the weekend. Among these ANE subcategories, the conducted statistical analyses based on the Kruskall–Wallis test to evaluate the variations of the individual SNRs of the events among the four pair combinations are significantly different (with *p* <0.01) for *bird*, *dog*, *door*, *peop*, *step* and *wrks* on the weekday and *bird*, *brak*, *dog*, *door*, *horn*, *peop* and *step* on the weekend day.

## 5. Discussion and Conclusions

In this work, we have advanced in the characterization of anomalous noise events in real urban environments, extending previous analyses by studying their presence (number of occurrences and total duration) and individual features (median duration and median SNRs) according to their evolution throughout the day and night, together with their local particularities found in narrow and wide streets during a weekday and a weekend day.

To that effect, a WASN-based database of 151 h has been designed and developed to have enough representative samples of this kind of acoustic events in real-life urban environments through the 24-nodes WASN of the DYNAMAP project deployed across District 9 of Milan pilot area. The conducted analyses show the regular presence of ANEs throughout the day in all sensed locations, besides confirming the unbalanced nature of the problem at hand, as the developed urban WASN-based database is composed of 126 h and 43 s of RTN passages (83.5%), 13 h 8 min and 48 s of ANE samples (8.7%), classified in 26 subcategories, 11 h 50 min and 29 s labelled as CMPLX (7.8%). Specifically, urban ANEs are mainly derived from the presence of pedestrians (e.g., *peop* and *step*), animals (e.g., *bird* and *dog*), transports (e.g., *airp*, *door*, *brak*, *sire*, *horn*) and industry (e.g., *wrks* and *inte*).

When comparing these results with the ones obtained from the preliminary manual recording campaign [28], substantial differences have been found. First, it is worth mentioning that in the WASN-based database the total amount of labelled ANEs (8.7%) is quite lower than the corresponding percentage observed in the manual dataset (12.2%). This result confirms the need of extensive recordings to characterize the urban environment properly—a similar conclusion was obtained when comparing the WASN-based suburban database with the previous manual recordings (1.8% and 3.2% of ANEs were found, respectively) [46]. Second, it is also worth noting that up to 11 non-previously observed urban ANE subcategories have been identified thanks to the WASN-based recording campaign. In particular, the annotators have completed the initial list of subcategories with *alrm*, *bell*, *blin*, *glas*, *inte*, *rain*, *rubb*, *sqck*, *step*, *trll* and *wrks*, being some of them quite predominant in the database. Finally, it should be pointed out that the WASN-based database is around 33 times larger than the expert-based dataset (of 4 h and 24 min of data, also composed of 20 minutes of continuous audio clips), as it was gathered in only 12 city locations of Milan at the street level during non-sequential diurnal periods (containing only one nocturnal recording).

Regarding the comparison between the urban and suburban WASN-based databases (both composed of more than 150 h of annotated environmental acoustic data), several issues can be discussed. First, the presence of ANEs in the urban environment is significantly higher than in the suburban counterpart (located at the A90 motorway surrounding Rome). The latter is composed of only 16 ANE subcategories (with 3,170 occurrences and 10,752.9 s of total duration [46]), being the most frequent ones *rain* and *thun* due to a fortuitous long thunderstorm episode that coincided with the weekend recordings. These events are followed by sounds derived from transports, like *brak* or *tran*, and animals (in this case, only *bird*) (see [19] for further details). Although meteorological ANEs have also been observed in the urban environment (i.e., *rain*, *thun*, and *wind*), their relevance is significantly lower as they have seldom been recorded. Nevertheless, this kind of events should also be discarded from the WASN-based measurements of noise levels due to their potential impact on the computation of $L_{Aeq}$ values as stated in both environments.

In what concerns the day–night plus narrow–wide pair combinations, both axes of the analysis methodology (see Figure 2) entail significant differences on the presence of ANEs for both recording days. In particular, the results have shown the major dependence of the presence of ANEs with respect to the time period axis than the street type axis. That is, ANEs have been mainly found (both in terms of number of occurrences and total duration) in day-time during the weekday, despite they have also been more observed in narrow than in wide streets, probably due to several issues such as the pass-by nature of RTN and the shorter distance between the source and the measurement point located at building facades that could entail higher SNRs, for instance. However, these preliminary considerations should be analysed in detail in future works, taking into consideration urban spatial features such as the height of buildings, the presence of furniture and greenery, or the so-called canyon effect in narrow streets [16,17], among others. Furthermore, it is also worth mentioning that several ANE subcategories show a relevant temporal dependence (e.g., *peop*, *step* and *door* in the diurnal period, *bird* at night and *wrks* on the weekday), along with other events that present a clear relationship with the type of street where have been found (e.g., *tram* and *brak* in wide streets or *step) in narrow streets*. When analysing the distribution of ANEs between day and night, most events have been found during the diurnal period, for both days, showing the weekend day a lower presence of ANEs both in terms of mean number of occurrences and total duration. However, several events are more present in the nocturnal period, such as *bird* and *inte*, when lower background RTN levels are typically measured. A similar pattern has been also found when comparing the results between narrow and wide streets, showing the former a higher presence of anomalous events. In what concerns the median duration of the ANE subcategories, we have found that most events show quite similar median durations regardless of the considered period of time and type of street. However, it is to note that events such as *bird*, *horn*, *sire* or *tram* present significantly lower durations in narrow than wide streets, besides other events such as *door*, *peop* and *step*, show also significant differences among the four configurations, together with events like *inte*, *musi*, *rubb*, *rain tram* and *wrks*, despite part of the latter group can be caused by the particular local nature of the events. On the other hand, median SNRs computed during the nocturnal periods from both days present higher values than in the diurnal counterparts (e.g., see *alrm* and *sqck*), which can be attributed to the lower background RTN levels, showing *bird*, *dog*, *door*, *peop*, *step* and *wrks* statistically significant differences among the four combination pairs. This issue may result particularly relevant in terms of the impact of the ANEs in the computation of the $L_{Aeq}$ values at night, as the permitted levels defined by the competent authorities are significantly lower than the corresponding ones for the day-time [10].

Future work will focus on advancing the research about the characteristics of real acoustic environments in two main directions: first, by designing and developing a complete spatio-temporal analysis of the ANE subcategories collected in both urban and suburban environments through both WASN-based recording campaigns, completing the study by considering complex audio passages, the effect of urban shapes and other street clusters; and second, by training the ANED algorithm with both WASN-based acoustic databases to improve its performance in real-operation environments.

## Figures and Tables

**Table 1:** Description of the sensors’ location across District 9 of Milan as the pilot urban area of the DYNAMAP project  [38], differentiating between those located in narrow streets (1 lane) and wide streets (more than 1 lane) by means of a horizontal line. X-lane/Y-lane street stands for a two-way street that has X lanes in one-way and Y lanes in the opposite way. X-lane street stands for a street with X lanes in the same way.

Sensor Id	Sensor location description
**hb115**	1-lane street with shopping in front
**hb133**	1-lane street, residential area, no shops, little park area in front
**hb135**	1-lane street with connection with 1-lane street (low speed), near University campus (students), no shops, in front of park area
**hb138**	1-lane street near connection with other 1-lane street, no shops
**hb139**	1-lane street, residential area, some shop/enterprise
**hb144**	1-lane street in residential area, one shop far away
**hb145**	1-lane street, in front of park
**hb124**	1-lane street, no shops
**hb125**	1-lane street with connection with 1-lane/1-lane street, mix of residential with some shops
**hb127**	1-lane street near bifurcation with 1 line street, some shop nearby
**hb137**	1-lane street with connection with 1 line street, in front of park, residential area, no shops
**hb106**	1-lane/1-lane street with connection with 1 line street, area with parks nearby, no shops
**hb136**	1-lane/1-lane street with connection with 1-lane street, area with parks nearby, no shops
**hb120**	1-lane/1-lane street, residential area, no shops
**hb151**	1-lane/1-lane street, bike lane, some shop and restaurant
**hb129**	1-lane/1-lane street, bike line, connection with 1-lane street, some shop
**hb108**	1-lane/1-lane street, in front University exit, no shops
**hb116**	1-lane/1-lane street with connection with 1-lane street, residential area
**hb114**	2-lane/2-lane street with shopping and business area and traffic light nearby
**hb121**	2-lane/2-lane street, connection with 1-lane street, University area, no shops
**hb140**	2-lane/2-lane street with parking area and traffic light with crossing nearby, no shops near and high traffic
**hb123**	2-lane/2-lane street with hotel and traffic light nearby
**hb117**	3-lane/3-lane street, near school, area with parks nearby, no shops
**hb109**	3-lane/3-lane street, near crossing with tramway and 1-lane+2-lane/2 lane+ 1-lane street, shopping and coffee/restaurant area

**Table 2:** Description of the 26 sound subcategories of anomalous noise events, in alphabetical order, identified during the manual labelling process of the WASN-based urban acoustic database, showing the number of occurrences, their total duration (in s), median duration (in s) and median signal-to-noise ratio (SNR) (in dB).

Label	Description	Total Occur. (#)	Total Dur. (s)	Median Dur. (s)	Median SNR (dB)
*airp*	Noise of airplanes and helicopters	250	3441.7	9.3	0.49
*alrm*	Sound of an alarm or a vehicle beep moving backwards	76	307.7	2.8	0.92
*bell*	Church bells	311	1142.2	1.4	2.62
*bike*	Sound of bikes and bike chains	943	1843.0	0.7	0.33
*bird*	Birdsong	4215	3632.3	0.6	0.47
*blin*	Opening and closing of a blind	4	20.5	2.9	6.13
*brak*	Brakes and conveyor belts	3245	5054.9	1.1	0.16
*busd*	Opening bus door (or tramway), depressurized air	277	218.0	0.7	0.65
*dog*	Barking of dogs	649	637.5	0.6	4.56
*door*	Closing doors (vehicle or house)	3843	2096.7	0.5	2.02
*glas*	Sound of glass crashing	35	31.5	0.8	2.84
*horn*	Horns of vehicles (cars, motorbikes, trucks, etc.)	957	954.3	0.8	2.18
*inte*	Interfering signal from an industry or human machine	52	2703.6	20.5	−0.74
*musi*	Music in car or in the street	146	984.9	1.9	0.48
*peop*	Sounds of people chatting, laughing, coughing, sneezing, etc.	5822	9452.7	0.8	1.82
*rain*	Sound of heavy rain	100	1060.6	6.2	−4.64
*rubb*	Rubbish service, sound of engine taking the container, emptying it and dropping it down	16	423.3	7.1	1.77
*sire*	Sirens (ambulances, police, etc.)	194	3980.3	9.67	0.59
*sqck*	Squick sound of door hinges	216	293.6	0.9	0.92
*step*	Sounds of steps	3574	2162.8	0.1	1.19
*thun*	Thunderstorm	1	1.0	0.99	5.71
*tram*	Stop, start and pass by sounds of tramways	185	1362.7	6.4	3.23
*tran*	Sound of trains	9	73.3	7.3	2.06
*trll*	Sound of wheels of suitcases (trolley)	251	1153.5	2.1	1.24
*wind*	Noise of wind (movement of the leaves of trees,...)	8	72.6	2.1	−0.43
*wrks*	Works in the street (e.g., saws, hammer drills, etc.)	1045	4222.9	1.5	1.27

**Table 3:** Mean number of occurrences and total duration per hour obtained for the weekday and weekend day, distinguishing between day and night-time periods, and being the diurnal period defined as 06:00–22:00 [29], and averaging the features by the number of sensed hours per period.

Recording Day	Mean Occurrences Per Hour (#)		Mean Total Duration Per Hour (s)	
	*Day*	*Night*	*Day*	*Night*
**Weekday**	5204.1	2718.0	9984.1	4427.2
**Weekend**	4308.0	2025.0	7159.1	3701.0

**Table 4:** Mean number of occurrences and total duration per street obtained for weekday and weekend day, distinguishing between narrow and wide streets (see Table 1), being the diurnal period defined as 06:00–22:00 [29].

Recording Day	Mean Occurrences Per Street (#)		Mean Total Duration Per Street (s)	
	*Narrow*	*Wide*	*Narrow*	*Wide*
**Weekday**	768.9	609.1	1,574.0	990.4
**Weekend**	594.0	342.3	1,037.8	550.3

**Table 5:** Mean number of occurrences and total duration per hour obtained for the weekday and weekend day, distinguishing between day- and night-time periods, being the diurnal period defined as 06:00–22:00 [29], and averaging the features by the number of sensed hours per period and number of streets per type.

Recording Day	Mean Occurrences Per Hour and Street (#)				Mean Total Duration Per Hour and Street (s)			
	*DR*	*NR*	*DW*	*NW*	*DR*	*NR*	*DW*	*NW*
**Weekday**	256.5	127.8	198.6	109.3	537.3	240.2	339.5	148.7
**Weekend**	235.2	123.6	143.4	55.4	405.7	226.5	224.7	100.8

## Abbreviations

The following abbreviations are used in this manuscript:

ANE	Anomalous Noise Event
ANED	Anomalous Noise Event Detector
CNOSSOS-EU	Common Noise Assessment Methods in Europe
CMPLX	Complex Audio Passage
DN	Day–Night Analysis
DR	Day–Narrow Analysis
DW	Day–Wide Analysis
DYNAMAP	Dynamic Noise Mapping
END	European Noise Directive
IoT	Internet of Things
NR	Night–Narrow Analysis
NW	Night–Wide Analysis
RTN	Road Traffic Noise
RW	Narrow–Wide Analysis
SNR	Signal-to-Noise Ratio
SPL	Sound Pressure Level
WASN	Wireless Acoustic Sensor Network
